# Living on the edge: Assessing the diversity of South African *Pocillopora* on the margins of the Southwestern Indian Ocean

**DOI:** 10.1371/journal.pone.0220477

**Published:** 2019-08-02

**Authors:** Brent Chiazzari, Hélène Magalon, Pauline Gélin, Angus Macdonald

**Affiliations:** 1 School of Life Sciences, University of KwaZulu-Natal, Westville, KwaZulu-Natal, South Africa; 2 UMR ENTROPIE (Université de La Réunion, IRD, CNRS), Laboratoire d’excellence CORAIL, Faculté des Sciences et Technologies, La Réunion, France; Academia Sinica, TAIWAN

## Abstract

Scleractinia of the Maputaland reef complex (MRC) in South Africa exist at the margins of the Western Indian Ocean (WIO) coral distribution and are the only substantial hermatypic coral communities in South Africa. *Pocillopora* species occupy a conspicuous component of the MRC, and previous investigations identified three species of *Pocillopora* utilizing conventional taxonomy. Thus, our aims were four-fold: to elucidate *Pocillopora* species diversity using genetic techniques, primarily using species delimitation methods based on the ORF gene; to test for the presence of hybridisation within the *Pocillopora* community on the South-West margin of distribution in the Indian Ocean using two nuclear and two mitochondrial markers; to test the presence of cryptic species, using 13 microsatellite markers, finally, to elucidate the degree of genetic diversity within each *Pocillopora* species found and compare this to communities in lower latitudes. We illustrate taxonomic inconsistencies between these inventories and our phylogenetic data. The MRC harbours unique populations of *Pocillopora*, consisting of three species hypothetically co-occurring throughout the south WIO, namely: *P*. *meandrina*/*P*. *eydouxi*, commonly misidentified as *P*. *verrucosa*, *P*. *verrucosa*, sometimes correctly identified, but also commonly misidentified as *P*. *damicornis sensu lato*, and *P*. *villosa*, almost always misidentified as *P*. *eydouxi*. The hypothesis that hybrid swarms of *Pocillopora* occur in marginal environments such as the MRC was not supported, with low levels of introgressive hybridization reported instead. Analyses illustrate low genetic diversity at the species and population resolutions, suggesting a small founder population for each species. Nevertheless, these populations are demographically unique, exhibiting high levels of ITS2 haplotype endemism compared to higher latitude populations and the rest of the WIO. *Pocillopora* diversity on the MRC represents a unique assemblage and warrants further protection.

## Introduction

Modern developments in phylogenetics and the development of genetics-based methods for delineating Scleractinians has improved our understanding of metazoan phylogeny [1]. *Pocillopora* species are characteristically plastic in colony morphology [2] and have historically challenged taxonomists [3]. Indeed, using morphological characters alone, notably the use of corallum macromorphology has proved troublesome [4] and as such, the use of genetics-based methods is useful to align species identification. Correlating genetic signatures to morphology in *Pocillopora* was proved difficult due to incongruencies between whole colony morphology and genetic lineages [5]. Some doubts have been expressed about the sole use of either of these techniques [6]. Nevertheless, research has aimed to reconcile phenotypic and genotypic traits with success using micromorphometrics (columella proportions) and genetic data in the *Pocillopora damicornis* complex [7,8]. These genetic studies have identified high species diversity in the *Pocillopora* genus; however, they have also highlighted that there may be no practical identification of these plastic species with grading morphs *in situ*.

Some studies [4,5,9] avoided primary morphometric species delineation and focussed on applying contemporary genetic algorithms without *a priori* species designations. These approaches have identified new cryptic lineages from different global biogeographic ocean provinces and linked conspecifics, primarily identified and grouped by morphology [3,7,9–13], though most of these studies were undertaken in isolation. In addition, the fast-paced identification and proposal of new species, the revival of junior synonyms and possible splitting of regional conspecifics, have all contributed to the knowledge about *Pocillopora* lineages and diversification. It has also demonstrated possible inconsistencies in nomenclature and allowed for taxonomic congruency of newly recognised genetic strains between studies. Using methodologies previously outlined [14], original and previously published mitochondrial and nuclear data were synthesized into haplotype collections without a primary focus on species membership [4]. The use of multiple markers and methods to assign Primary Species Hypotheses (PSH), and the addition of further analyses (microsatellite markers) to assign Secondary Species Hypotheses (SSH), form a robust basis for delimitation of true evolutionary units. These methods and the naming and cataloguing of the genetic diversity of the *Pocillopora*, provide a collated reference dataset of genetic marker information on the phylogenetics of *Pocillopora* including microsatellite data. This accessioned dataset provides a powerful referencing tool for aligning localised genetic diversity of *Pocillopora*, even if species designations are not yet known, so as to avoid pre-emptive designations based on morphology alone.

Scleractinia of the Maputaland reef complex (MRC) in South Africa exist at the margins of the Western Indian Ocean (WIO) reef coral range and are the most diverse hermatypic coral communities in South Africa. These coral communities are linked to the WIO centre of diversity by Mozambican Channel eddies that move warm water southward from the Equator transporting coral larvae southward [15]. This and the favourable stable East African marine environment may account for the comparatively diverse coral assemblages found on this high latitude reef environment. *Pocillopora* species occupy a conspicuous component of the MRC, covering about 1% of total reef cover, with hard coral abundance having increased in the last 15 years [16]. The diversity of South African hermatypic corals has been previously investigated [17,18], and they identified three species of *Pocillopora* (*P*. *damicornis*, *eydouxi*, and *verrucosa*) using conventional taxonomy (i.e. based on morphological criteria). However, these communities have yet to be investigated using molecular methods, which would improve the understanding of these corals. We predicted that evolutionary novelties could occur within this marginal reef assemblage where, for example, hybridisation may favour adaptation in this marginal environment [19–22]. It has been reported that genetic structuring of two *Acropora* species occurring in the MRC indicates a bidirectional flow of genetic diversity [23,24] as is the case of other species’ passive dispersal against the Agulhas Current possibly due to strong nearshore counter currents and other oceanographic phenomena [25]. These phenomena could have an effect on the perceived southward attenuation in genetic diversity in *Pocillopora*.

Species delineation of *Pocillopora* using genetics has improved in the past few years, with novel and complete datasets [3–5,7–9,11,12,26–30], and methods available for scientists to use [3–5,7,9–12,29,31–34]. Using these methods, the objective was to produce DNA sequences for the mitochondrial ORF and D-loop, the nuclear HSP70A and ITS2 and microsatellite data, *a priori* of morphological designation. Our aims were four-fold: First; to elucidate *Pocillopora* species diversity, primarily based on the ORF gene defining Primary Species Hypotheses (PSH), second; to test for the presence of hybridisation within the *Pocillopora* community on the South-West margin of distribution in the Indian Ocean using two nuclear and two mitochondrial markers, third; to define Secondary Species Hypotheses (SSH) and the presence of cryptic species, using 13 microsatellite markers, and finally forth: elucidate the degree of genetic diversity within each *Pocillopora* species found using all markers. These data are compared to already collated datasets, thus allowing investigation of the unique position and structuring of the MRC *Pocillopora*. We hypothesise that the MRC may hold similar levels of genetic diversity for the *Pocillopora* as lower latitude reef systems of the WIO region and harbour unique lineages new to science. Here we present an investigation into the diversity of *Pocillopora* using contemporary markers and techniques.

## Materials and methods

### Identification, collection, and preservation

**Fig 1:**
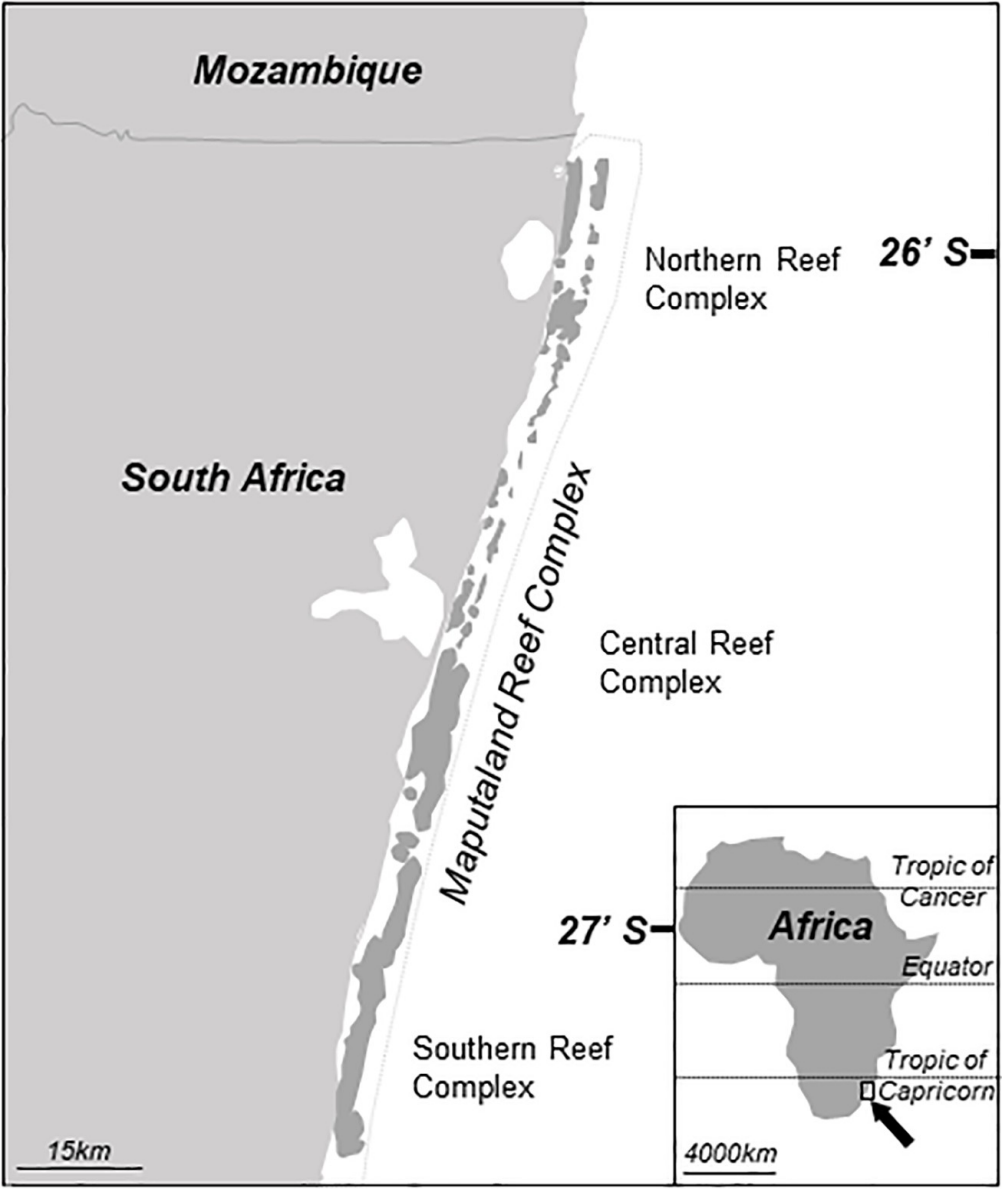
Map of the Maputaland Reef Complex within South Africa’s Isimangaliso Wetland Park from which all samples were collected.

Fifty-seven *Pocillopora* colonies were photographed and sampled from the Northern, Central, and Southern MRC within the Isimangaliso Wetland Park (Fig 1) covering a distance of 100 km, between 2014 and 2017 using the collection permits RES2012/66 and RES2014/06 from the South African Department of Forestry and Fisheries. Emphasis was placed on documenting as many colony forms as possible. To reduce the chance of collecting clonemates, only colonies that were more than 5 m apart were collected. Apical branches of *Pocillopora* colonies (~10 cm length) were collected using SCUBA and free diving from depths ranging from 0 to 35 m and encompassing all reef habitats. Finally, colony fragments were stored at room temperature in 100% ethanol for processing.

Each colony was assigned to a morphotype (named numerically) based on corallum macro-morphology using *in situ* photographs, according to widely accepted descriptions [6]. Using colony form as a guideline, and through personal communication and inspection with a previous checklist author (Dr Louis Celliers) and coral field-expert (Dr David Obura), colonies were compared and aligned with the morphotype and species description of previous checklists focused on the MRC [17,18].

### DNA extraction, PCR amplification, and sequencing

Whole genomic DNA was extracted from each colony using a Zymogen Quick-gDNA MiniPrep extraction kit following overnight proteinase-K digestion, according to the manufacturer’s protocol (Zymo Research Corporation, California, USA). PCR was conducted to amplify the Open Reading frame (ORF) amplified using the FATP6.1 and RORF primers [31], the putative control region (CR) in the mtDNA D-loop structure using the FNAD5.2deg and RCOI primers [31], Heat Shock Protein (HSP70A) using the HSP70A-F and HSP70A-R primers [10], and the Internal Transcribed Spacer region 2 (ITS2) using the ITSc2-5 and R28S1 primers [31].

PCRs were performed in 25 μL of 1X OneTaq Quick-Load (New England Biolabs) 1X Master Mix, 0.3 μM of both forward and reverse primers, 1 μM of bovine serum albumin (BSA), 2–20 ng/μL of extracted DNA template. The PCR thermal cycle was: [95°C for 5 min], 30 × [(94°C for 30 s) (55°C for 45 s for ORF, 58°C for 45 s for D-loop, and 58°C for 45 s for ITS2) (72°C for 45 s)] and [72°C for 10 min], [4°C for ∞]. PCR product for each sample was checked for quality and quantity using a Nanodrop 3000 spectrophotometer and subsequently run on a 1% agarose gel, using 1 μL of loading dye and 5 μL of PCR product per sample. Samples were then sequenced using an ABI 3730 capillary sequencer using Big End Dye technology at Inqaba Biotechnical Industries (Pty) Ltd, Hatfield, South Africa.

Colonies were further genotyped using 13 microsatellite loci: Pd2-001, Pd3-004, Pd3-005, Pd2-006, Pd3-008, Pd3-009 [35], PV2, PV7 [36], Poc40 [9], Pd4, Pd11, Pd13 [37] and Pd3- EF65 [38]. Forward primers were indirectly fluorochrome labelled (6-FAM, VIC, NED, PET) by adding a universal M13-tail at the 5’-end (5’-ACGACGTTGTAAAACGAC-3’) [39] and were multiplexed post-PCR. Each amplification reaction was performed in a total volume of 10 μL: 1X of MasterMix Applied (Applied Biosystems), 0.025 μM of forward primer tagged with the M13-tail, 0.25 μM of reverse primer, 0.25 μM of fluorescent dyed M13-tail and 2 ng/μL of genomic DNA. The thermocycling program was: [94°C for 5 min], 7 × [94°C for 30 s, 62°C (- 1°C at each cycle) for 30 s, 72°C for 30 s], 30 × [94°C for 30 s, 55°C for 30 s, 72°C for 30 s], 8 × [94°C for 30 s, 56°C for 30 s, 72°C for 30 s] for the final hybridization of fluorescent dyed M13-tail + 72°C for 5 min. PCR products were genotyped using an ABI 3730XL sequencer at the Plateforme Gentyane (INRA, Clermont- Ferrand, France). Allelic sizes were determined with GENEMAPPER 4.0 (Applied Biosystems) using an internal size standard (Genescan LIZ-500, Applied Biosystems).

### Sequence editing and phylogenetic analyses

Sequences were checked, edited and aligned using Bioedit 7.2.6 [40]. A collated list of haplotypes of the ORF (55 haplotypes), D-loop (32 haplotypes), and ITS2 (128 haplotypes) genes [4], were used as a reference for comparison to local South African congeners. ORF has consistently demonstrated suitability in defining species boundaries [3–5,11,34], thus we present our PSH findings primarily based on the ORF gene. The remaining genes were used in conjunction with the ORF gene, mostly for corroboration to describe new genetic lineages of the genes within the corals studied.

The ITS2 sequences were considered autologous and double peaks were resolved with reverse sequences and then by using PHASE 2.1.1 [41]. ITS2 haplotype sets were reconstructed for all sequences using Phase 2.1.1 with 100 burn-in iterations, followed by 100 iterations, all with different random starting seeds and a threshold probability of 0.9. Haplotype sets were constructed in DnaSP v6 [42], and final ITS2 haplotype sets all had single nucleotide polymorphisms (SNPs) of probability 1.0. Gaps in all sequence sets were counted and all variable sites were used.

MrModeltest [43] was used to determine the appropriate substitution model of evolution for each gene (ORF: K80, CR: K80, ITS2: K80, HSP70A: HKY) for Bayesian phylogenies using MrBayes [44]. All haplotypes were assigned the same weight. The Bayesian trees constructed with haplotypes were created using four Markov Chain Monte Carlo (MCMC) iterations of 20×10^6^ generations each, sampled every 10 generations. The first 20×10^3^ trees were discarded as burn-in, with the rest of the genealogies used to construct a 50% majority-rule consensus tree. Ultrametric phylogenies using BEAST 2.0 were completed using the GTR+I+G model for each marker [45]. Four MCMC chains were run for 20×10^6^ generations and sampled every 20×10^3^ generations. Parameter convergence (ESS values were at least 800 for all trees) was verified using Tracer [46], and tree convergence topology was checked using AWTY [47] for all Bayesian analyses. The consensus trees (maximum clade credibility tree; 10% burn-in) were constructed with TREEANNOTATOR 1.7 [48].

Maximum Likelihood (ML) methods were conducted using phyML [49], using the same substitution models calculated in MrModeltest [43], and results were used for the Automatic Barcode Gap Discovery (ABGD) species delimitation method by analysing the distribution of pairwise differences within a dataset. Trees were rooted using two sister genera within the Pocilloporidae; *Stylophora* [50] and *Seriatopora* [51], accession numbers: KX618661–KX618678 (for ORF, CR, and ITS2) and JX869141–JX869151 for HSP70A, after a BLAST search [52]. Bootstrap values greater than 75% and posterior probabilities of 0.95 were accepted as robust and included in trees [53]. Outgroups were pruned for species delimitation analyses and haplotype networks. Haplotype networks were created using PopART [54] using the minimum spanning network construction [55].

### Species delimitation

The methods used to delimit putative species boundaries for the ORF, D-loop, and HSP70A genes, were the Automatic Barcode Gap Discovery (ABGD [56]), Poisson Tree Processes model (PTP [57]), Bayesian PTP (bPTP), and the generalized mixed Yule coalescent model (GMYC; [58]). Each analysis employed equal weighting of one individual per haplotype, as this has been shown to give the most parsimonious and congruent results among delimitation methods [58]. The four species delimitation methods were used in combination to identify species boundaries using the PSH method, considering how often each method partitioned haplotypes into species. Fields for recombination (FFR) were used to distinguish species breaks using the ITS2 gene, following published criteria [59].

ABGD was used to develop genetic distance-based methods to compare non-overlapping intra and interspecific genetic distance, using the haplotype sets uploaded to the online server: http://wwwabi.snv.jussieu.fr/public/abgd/abgdweb.html. The priors set for each marker were the same: Pmin = 0.001, Pmax = 0.08 and X = 1.5, using the Kimura (K80-2P) model of evolution [60]. PTP methods were used to test the number of substitutions among leaves, where the probability of a substitution tested gives rise to a species, assuming a Poisson distribution. PTP and Bayesian implementation of PTP (bPTP) methods were carried out using the ML tree as per suggestions on the PTP online server: http://species.hits.org/ptp/ [57]. The bPTP analysis was run for 2×10^6^ MCMC generations and a burn-in of 5×10^5^, thinning every 200 generations, and convergence was checked visually as recommended [57]. GMYC methods were carried out using the GMYC online server: http://species.h-its.org/gmyc/ using previously described methods [61]. Ultrametric phylogenies were first calculated using BEAST 2 [45]. Priors were set in BEAUti [45] following a strict Yule process, i.e. no extinction using a relaxed log-normal clock and assuming a neutral coalescence tree [62]. Three MCMC iterations were run for 20×10^6^ generations and sampled every 2×10^3^ generations. Chain convergence was assessed using TRACER 1.6 [46] and tree convergence topology was checked using AWTY [47]. The bGMYC results were run using the cut-off probability of 80% [bGMYC (80)]. The consensus trees (maximum clade credibility tree; 10% burn-in; tree not presented) were constructed with TREEANNOTATOR 1.7 [48].

### SSH delimitation using microsatellite data

Genotyped individuals were assigned to an SSH with reference to a comprehensive published dataset [4]. The sixteen PSHs identified were used as a point of reference for species clusters in our data set. Population structure was assessed using Bayesian clustering analysis in the program Structure 2.3.4 [63]. The analysis was run for the whole dataset using five MCMC simulations per run, with 10^6^ iterations and 5×10^5^ steps as burn-in, to test possible clusters (K) between one and ten. Structure harvester was used to determine the most likely combination of homogenous clusters [64].

## Results

### Sequence variability and haplotype assignment

The fifty-seven colonies collected from the MRC yielded three ORF haplotypes (24 ORF27, 19 ORF46, 11 ORF39), of which three individuals did not amplify. These haplotypes are previously published [4], with 15 variable sites over 842 base pairs (bp) (π = 0.008) and a G+C content of 40.0%. The trimmed and aligned CR made up 464 bp of the D-loop region, beginning from position 441 bp of D-loop. The CR sequences yielded two unique haplotypes, dissimilar from haplotypes of previously published lists, due to a single adenine insertion at position 322. Disregarding this insertion, all but four individuals would belong to previously recorded D-loop04 (n = 19) and D-loop12 (n = 28) *sensu* [4]. The CR haplotypes varied over 9 sites of the 464 bp (π = 0.005) and a G+C content of 40.2%. ITS2 haplotypes consisted of 31 haplotypes with four previously published haplotypes and 27 new ones, and varied over 11 sites of the 710 bp (π = 0.003) with a G+C content of 57.6%. Nuclear HSP70A haplotypes varied over 9 sites of the 741 bp (π = 0.003) and had a G+C content of 48.7%, these sequences could not be compared to established lists due to their short length, compared to the whole HSP70 region (HSP70A + HSP70B). Forty-nine colonies had the CR and ORF, and HSP70A and ITS2 regions concatenated for analysis. The mtDNA (CR+ORF) sequences had 24 variable sites over 1724 bp (π = 0.0068), with a G+C content of 39.4%. The phased nDNA (HSP70A+ITS2) sequence set consisted of 52 reconstructed haplotypes, and 36 variable sites over 1230 bp (π = 0.0061), with a G+C content of 52.8%. Unique sequences obtained using the four genes were deposited into GenBank [65] with the accession numbers: ORF: MK359369—MK359422, ITS2: MK385611—MK385625 and MK359423—MK359433, CR: MK429833—MK429834, HSP70A: MK429806—MK429832.

### Mitochondrial DNA phylogenetic analysis and species delineation

**Fig 2:**
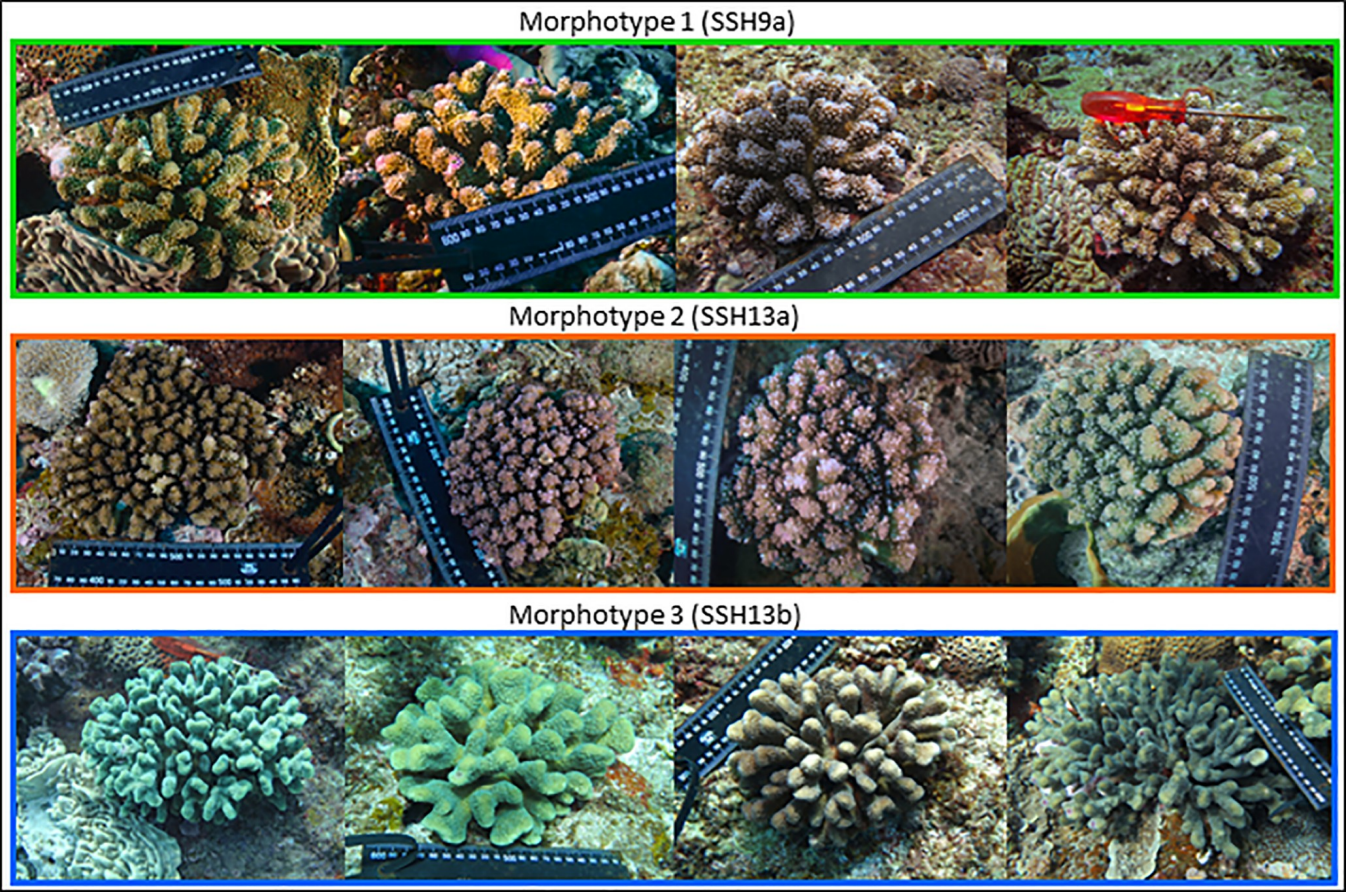
*In situ* photographs of the three colony morphotypes collected at the Maputaland reef complex, South Africa. Coloured borders correspond to the assigned Secondary Species Hypotheses (SSH) (green: SSH09a, orange: SSH13a, blue: SSH13b).

**Fig 3:**
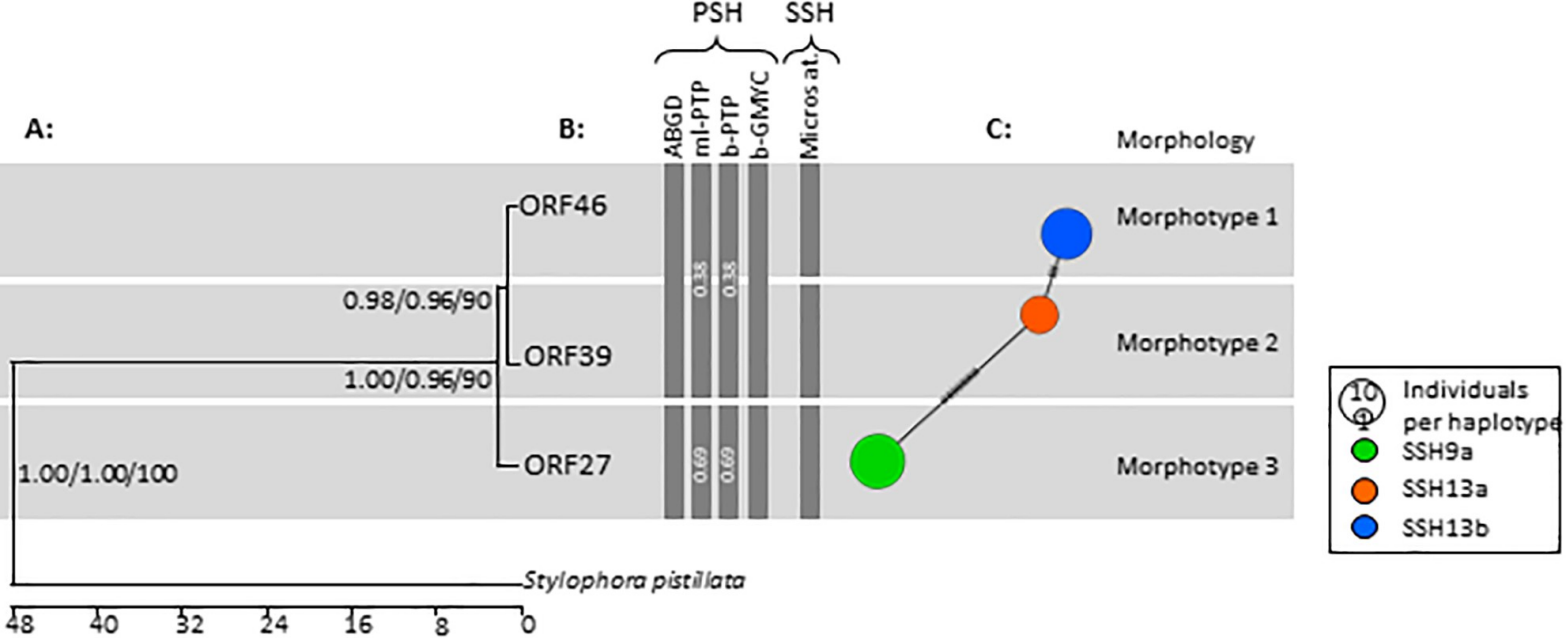
Phylogenetic analysis of *Pocillopora sp*. of South Africa in the Southwestern Indian Ocean (SWIO), using the mtDNA region: Open Reading Frame (ORF). (A) Ultrametric phylogram of the three ORF haplotypes. Node values indicate Ultrametric probability/Bayesian probability/Maximum Likelihood values. (B) Species delimitation methods using ABGD, ML PTP, B PTP, and GMYC methods using Primary Species Hypothesis (PSH) criteria, and Secondary Species Hypothesis (SSH) using thirteen microsatellite markers. (C) Haplotype network coloured according to PSH delineation and sized according to the number of colonies. Scale bar represents millions of years before present.

Bayesian, ultrametric and ML phylogenies for the ORF were congruent using single and multiple individuals per haplotype for the ABGD analysis when using GTR and HKY model parameters. The time-calibrated ORF phylogram formed three significantly distinct and robust clades, for each of the three haplotypes, and correlated with the *a priori* morphotypes (Fig 2). Calculated divergence time for ORF27 from ORF39 and ORF46 was approximately 2 million years ago (MYA) with a 13 bp difference, while ORF39 and ORF46 shared a divergence time of approximately 1.8 MYA (Fig 3) with a 3 bp difference. The control region (CR) data resulted in a similarly well-supported phylogram of three haplotypes, although it resolved weaker relationships between our *a priori* morphotype allocations and PSH than the ORF (S1 Fig). Divergence times using the CR were identical to the ORF. CR could not be compared to previous studies due to shorter sequence lengths in this dataset. Nevertheless, the 1 bp insert at position 322 is enough to determine that at least one unique D-loop haplotype exists in the studied colonies.

All delimitation methods resolved the same two putative species for the ORF gene: the first species corresponding to ORF46 and ORF39 colonies and the second one to ORF27 colonies. ABGD resolved these two clades, albeit with low statistical significance (p = 0.68). Similar results were obtained by employing GMYC methods. These PSHs were named in accordance with previous species delimitations [4]: PSH09 and PSH13. These PSHs show barcoding gaps with a comprehensive range of haplotypes not present in South Africa, and have further divisions corresponding to SSH using assignment tests with described microsatellite data [4]. Thus, the haplotypes in this study are represented with reference to these SSHs, namely SSH09a (ORF27), SSH13a (ORF39), and SSH13b (ORF46) to gain a global perspective. Here, the three haplotypes do not share any individuals in terms of assigned *a priori* morphotypes according to the network analysis. These results confirm the reproducibility of methods and congruency of results [4,33].

The division of ORF39 and ORF46 (corresponding respectively to SSH13a and SSH13b) was not clear using sequence-based delineation methods. However, the phylogram, sl-FFR, and microsatellite analysis showed a significant and congruent delineation of the SSH13a and SSH13b haplotypes. SSH identification using 13 microsatellite markers resulted in the delineation of SSH13a and SSH13b, corresponding with our three ORF haplotypes and the three *a priori* morphotypes (Fig 3B). Microsatellite analysis resulted in three clusters for individuals of ORF27 (SSH09a); namely SSH09a-1, -2, and -3 [33]. These three clusters fell within the SSH09a delineation and showed no clear discerning colony morphology within SSH09a.

CR data (S1 Fig) were shorter in length and were congruent with the results of the ORF gene. The concatenated mtDNA (ORF+CR) resulted in five haplotypes (S2 Fig). This composite sequence set could not be compared to existing datasets due to the shorter CR length. Nevertheless, this dataset corroborates the ORF data. Thus, we only present the results from the ORF; CR and the mtDNA analysis in the supplementary information.

### Nuclear DNA phylogenetic analysis

The nuclear HSP70A data were congruent with the ITS2 dataset, even though only half the HSP70 gene was used (S2 Fig). The concatenated nDNA (ITS2+HSP70A) dataset resulted in 56 haplotypes (S1 Fig). These data were not included in our argument as they were mostly congruent with the primary data (the ORF and ITS2 gene, and the microsatellite data). These data were excluded as they were mostly not comparable, nor were they used consistently across previous studies. Thus, for the sake of continuity and reducing redundancy, we report these results as a supplementary addition to the study.

SSH09a was composed of 16 ITS2 haplotypes (two shared: one each with SSH13a and SSH13b). SSH13a was composed of six haplotypes (one shared with SSH09a). SSSH13b was composed of 10 haplotypes (one shared with SSH09a). Ten of the ITS haplotypes (Haplotypes ITS2-129 to ITS2-138) were novel. Four previously discovered haplotypes are present in South African populations: ITS2-002, ITS2-009, and ITS2-014 *sensu* [4]. The ITS2-014 haplotype was only associated with individuals of SSH09a for the first time. The ITS2-014 haplotype has only previously been reported [4] in association with ORF39 (SSH13a) and ORF47 (SSH13b).

**Fig 4:**
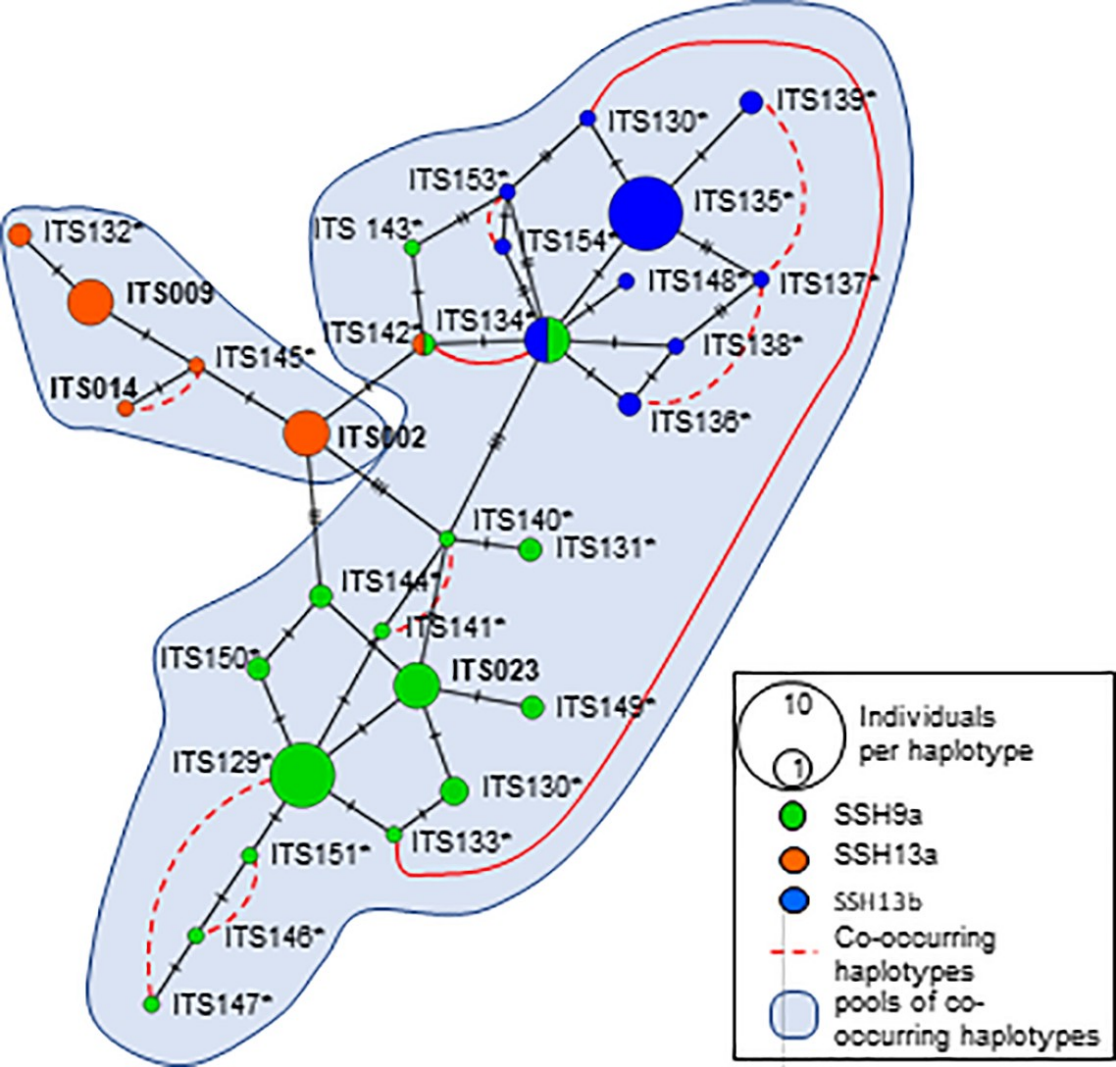
Internal transcribed spacer 2 (ITS2) haploweb of *Pocillopora* diversity of South Africa in the Southwestern Indian Ocean (SWIO), constructed using minimum spanning. Each haplotype is sized according to the number of colonies and coloured according to the Secondary Species Hypothesis (SSH). Red dashed curved lines represent haplotypes found co-occurring in heterozygous individuals (solid lines indicate co-occurring haplotypes across SSHs). Haplotypes marked with an asterisk indicate newly discovered ITS2 haplotypes, and haplotypes in bold are previously reported in the literature.

ITS2 phylogenies were mostly congruent with the ORF data for the three major clades but with weaker support, more phylogenetic structure, and the mismatch assignment of some individuals to different clades. The tree topology can be attributed to a large number of haplotypes, the polymorphic nature of the nuclear ITS gene, and the possibility of hybridisation within the *Pocillopora* complex. Thus, due to its congruency with the ORF gene, these results are not presented here. The network analysis (Fig 4) revealed a complex of mostly homogeneity in colony form within the SSHs (based on ORF and microsatellite assignment tests) with two haplotypes consisting of more than one *a priori* morphotype (ITS2-134 and ITS2-142). Ten single locus co-occurring haplotypes were present (red dashed lines, Fig 4), two of which occur across PSH and SSH boundaries (solid red line, Fig 4). These co-occurring haplotypes delineate two pools of sl-FFR (Single locus fields for recombination); the SSH09a and SSH13b group, and the second pool composed of only SSH13a, SSH13a and SSH13b shared no sl-FFR.

### Coral morphology and species identification

Coral colonies were classified into three *a priori* morphotypes using previous descriptions [6] and these related to the morphological species descriptions listed in the two South African checklists [17,18]. Morphotypes 1, 2, and 3 were associated with the species delineation as discussed in the previous paragraphs, namely; species SSH09a, SSH13a, and SSH13b. Thus, each ORF haplotype and corresponding SSH could be matched with species of previous checklists; SSH09a related to *P*. *verrucosa*, SSH13a related to *P*. *verrucosa* and *P*. *damicornis*, and SSH13b related to *P*. *eydouxi*; according to the descriptions of previous South African checklists [17,18]. The three SSHs were then correlated to current accepted morpho-genetic species designations from contemporary literature [5,7,8,10,11,26,27,29,30,66] and a collated study including all ORF haplotypes [4]; SSH09a related to *P*. *eydouxi/meandrina*, SSH13a related to *P*. *verrucosa*, and SSH13b related to a distinct species, sometimes identified as *P*. *verrucosa*, but for which the name *P*. *villosa* has been proposed if no authority already exists [4].

Thus, *P*. *verrucosa* previously described and listed in South African waters belong to SSH09a (ORF27) and appears to be congruent globally with the *P*. *eydouxi/meandrina* complex. *Pocillopora damicornis/verrucosa* previously described and listed in South African waters belongs to the *P*. *verrucosa* (SSH13a) species globally. Lastly, the *P*. *eydouxi* species described in South African waters belong to the morphologically and genetically distinct clade SSH13b, with a newly proposed name of *P*. *villosa* (if no authority already exists). These overall discrepancies between contemporary phylogenetics and morphological identification in the previous checklists [18,67] are evident, seem to be mostly incorrect but consistent, and are highlighted in Table 1.

**Table 1:** Relating DNA analysis to morphology and current literature and checklists, of South African *Pocillopora*.

Literature		This study				Contemporary Literature	
South African checklists		Morphology	DNA			Taxonomy	
Riegl (1996)	Celliers and Schleyer (2003)	*a priori* colony morphotype	Open reading frame Haplotype	Primary Species Hypothesis	Secondary Species Hypothesis	Individual studies	Collated literature [4]
*P*.* verrucosa*		Morphotype 1	[4]**; ORF27**[11]; A ][5,9]; 1a [7]; em[26]; N/A [27]; N/A [8]; IIb	PSH09	SSH09a-1 SS09a-2 SSH09a-3	[11] *eydouxi*, *meandrina* [5,9] *eydouxi*, *meandrina*, *verrucosa*, *damicornis* [7] *eydouxi*, *meandrina* [26] *meandrina* [27] *eydouxi*, *meandrina* [8] *eydouxi*, *meandrina*	*P*.* eydouxi*, *meandrina* complex in the Indian Ocean.
*P*.* verrucosa*, or *P*.* damicornis* (noticeably in young colonies).		Morphotype 2	[4]**; ORF39** (5, 10); 3c (27); NF	PSH13	SSH13a	[5,9] *verrucosa* (27) N/A	*P*.* verrucosa* of the Indian Ocean
*P*.* eydouxi*		Morphotype 3	[4]**; ORF46** [5,9]; 3a [7]; γ [11]; N/A [27]; N/A	PSH13	SSH13b	[5,9] *damicornis*, *verrucosa*, *meandrina* [7] *γ-verrucosa* [11] *molokensis* [27] N/A	Genetically distinct subclade in the *P*.* verrucosa* complex (known as the "doom-trap"). [4] a new name proposed; *P*.* villosa*

## Discussion

*Pocillopora* species from South Africa appear to belong to three commonly occurring species on the Maputaland reef complex (MRC), according to contemporary genetic delineation techniques; *Pocillopora eydouxi/meandrina* (SSH09a), *P*. *verrucosa* (SSH13a), and another genetically distinct species (SSH13b, and a proposed name of *P*. *villosa*). Morphological identification of South African *Pocillopora* seems to suffer from the same difficulties in identification as many regional studies from around the world. These designations are out of sync with contemporary phylogenetics which is now widely accepted. Previous South African checklists [18,67] and numerous studies employing the same taxonomic designations [16,68–72] may be affected. Each of the species identified in this study seems to be affected, and as such, we have tried to refrain from solely using previously accepted names, but also include the SSHs, which represent the genetic lineage of these species. Added to this, we do reconcile our genetic findings with previous checklists. This dynamic evolutionary environment suggests this marginal habitat–in terms of hermatypic coral assemblages–may harbour unique phylogenetic diversity and limited hybridisation amongst these species.

### Use of mitochondrial, nuclear, and microsatellite markers for *Pocillopora* in South Africa

Nuclear DNA data suggests a unique haplotype profile, but low diversity at the population level when compared with other lower latitude populations. Fifty-seven colonies of *Pocillopora* were sampled for species-level diversity in South Africa for the first time. The MRC marks the southwestern margin of hermatypic coral diversity in the WIO bioregion, where a paucity of research on coral biogeography exists. Recent insight [3] has highlighted the concordance of the ORF marker with holobiont, transcriptomic, SNP, and conspecificity data. Added to this, a collection of data now exists for the marker in the form of a collated list of haplotypes [4]. Thus, species assessment using the ORF marker was appropriate for delineation in South African *Pocillopora* and demonstrates the efficacy of this marker and suggests its use as a principal barcode marker. This data also corroborates and illustrates the reproducibility of our methods developed and employed by many studies over different regions [3,4,7,10,13,30,32–34,37].

The failure of the CR primers [11] to amplify the much longer CR gene across our dataset suggests mutations within the primer annealing sites. The CR exhibits phylogenetic congruency with the considerably shorter ORF gene. The ITS2 marker was easier to amplify than the HSP70A and B regions. Evidence of this was apparent by the high failure rate of the HSP70B marker in this study, and we were forced to use only half the gene region (HSP70A). Although the ITS2 gene has been reasoned to be a poor barcode marker, it may have merit in identifying population structure, paternal lineages, possible backcrossing, incomplete lineage sorting, and hybridisation events. Microsatellite data were able to resolve all species groupings with precision and congruency with the other marker sets. The combination of sequence-based species delineation, microsatellite genotyping, and FFR methods across multiple markers resolved reliable results that probably describe the species of *Pocillopora* in South Africa.

### Taxonomic assessment of *Pocillopora* in South Africa

South African *Pocillopora* records are at odds with those described by contemporary phylogenetic methods (Table 1). Nevertheless, South African checklists [17,18] are largely consistent with one another, allowing for easier identification of species and comparison with our phylogenies (Table 1). We identified three species in South Africa: *P*. *eydouxi/meandrina* (SSH09a) *P*. *verrucosa* (SSH13a), and the distinct newly proposed *P*. *villosa* (SSH13b). All the studied colonies assigned to characteristic SSH09a harbouring morphotype 1 (*P*. *meandrina/P*. *eydouxi* morphotype; Table 1), and corresponded to *P*. *verrucosa* in South African checklists [17,18]. Morphological similarities between the *P*. *meandrina/ P*. *eydouxi* complex and that of *P*. *verrucosa* may explain the incorrect taxonomic designation (see previously described similarities [2]). In addition, SSH09a colonies are phenotypically plastic which may blur species boundaries that have been based solely on morphology explaining why both *P*. *meandrina* and *P*. *eydouxi* names have been used. Nevertheless, the South African *P*. *eydouxi/meandrina* (SSH09a) population consists of one haplotype, ORF 27, and is regarded as a distinct species using our delineation methods. These species boundaries are supported by eight genetic studies and morphologically designated as *P*. *eydouxi* with a 75% frequency [3–5,7–9,11,29]. Morphologically, SSH09a does fit conventional descriptions of *P*. *eydouxi* [2,6]. However, novel research [3] using RAD-seq data found it is not possible to discern *P*. *meandrina* morphotypes from *P*. *eydouxi* using the ORF marker. In addition, our microsatellite genotyping revealed the presence of all three population clusters presently known to SSH09a (-1, -2, and -3, Table 1). It is hypothesised that these three clusters possibly reflect three cryptic species existing in sympatry in the WIO [33]. Recently, a method using the Histone-3 region has been successful in delineating the *P*. *eydouxi/meandrina* complex [3]. More investigation in this species complex is needed using this method as our study was not able to correctly discern the morphologies and genetics.

In a similar fashion to the *P*. *meandrina/eydouxi* (SSH09a) colonies, the MRC colonies assigned to SSH13b harboured a single ORF haplotype (ORF46; [4]) and were all assigned to morphotype 3 (Table 1). Although sequence-based delimitation methods failed to identify the SSH13b clade as a species, phylogenetic and microsatellite data support this unique clade (Fig 3). Assignment tests [4] based on microsatellite data that this unique haplotype, with little interspecies genetic divergence, exists in sympatry with SSH13a and SSH13c. Together with its easily identifiable and unique colony morphology, and the argument that SSH13b should constitute its own species; researchers [4] proposed the name *P*. *villosa* due to its particular and recognizable velvety aspect. The SSH13b morphological description matches in some aspects to the description [6] of *P*. *elegans* and *P*. *eydouxi*, and has been previously identified [17,18] as *P*. *eydouxi* in South African checklists (Table 1). We believe the argument that SSH13a and SSH13b represents one morphologically plastic species is unlikely. The evidence against this possibility includes a unique genetic identity across all of the markers tested with unique monophyletic and statistically robust clades, no evidence of shared genetic material (haplotypes) or hybrid backcrosses. Thus, we conclude that *P*. *villosa* (SSH13b) should constitute its own species.

*Pocillopora*. *verrucosa* (SSH13a) colonies from the MRC also harboured a single ORF haplotype (ORF39) and they were all assigned to a unique morphotype (morphotype 2, i.e. *P*. *verrucosa* morphotype; Table 1), which were identified as *P*. *verrucosa* and *P*. *damicornis* by Riegl [17] and Schleyer and Celliers [18]. Contrary to SSH09a and SSH13b corresponding to a unique species in the various checklists, *P*. *verrucosa* (SSH13a) corresponds to two species: *P*. *verrucosa* and *P*. *damicornis* (*sensu lato*) in the South African checklists. Indeed, *P*. *damicornis* (*sensu lato*) is reported in both Scleractinian checklists in South Africa (Table 1). Yet, after a search for unique colony morphotypes, including many individuals that may resemble *P*. *damicornis-*like colonies, no colonies harbouring the corresponding ORF haplotypes were collected (haplotypes ORF01 –ORF21, species PSH01 –PSH05). It is possible that young colonies of other *Pocillopora* growing in marginal habitats (such as rock pools of intertidal zones) are mistaken for *P*. *damicornis*. Indeed, these colonies sometimes show finer branching where verrucae grade into branches and may resemble true *P*. *damicornis*. Interestingly though, researchers [69] report *P*. *damicornis* on Nine Mile Reef on the MRC, which possibly suggests healthy, phenotypically representative adults of *Pocillopora* sp. were mistaken as *P*. *damicornis (sensu lato)*. Others [68] also report the coral’s presence in most tidal pools in the MRC and as far south as Isipingo, KwaZulu-Natal. Having sampled some of the exact localities of these studies, it is unlikely that we did not account for these morphotypes.

Incorrect morphological identification of small and finer branched colonies belonging to SSH13a (associated with the *P*. *verrucosa* morphotype) is the most likely case for misidentification of *Pocillopora* in South Africa. The results of our study illustrate the difficulty in identification of cryptic lineages without corroboration employing genetic analysis. It is also clear that certain instances of identification in various other studies were incorrect, owing to the varied morphological assignments in each haplotype, a probable artefact of misidentification [4].

### Genetic diversity and structure of South African *Pocillopora*

Analyses suggest low genetic diversity at the species and population resolutions, as evidenced by the low haplotypic diversity and lack of linkages in the haplotype network (Fig 3). South Africa harbours three *Pocillopora* species [possibly five, considering the three diverging clusters within SSH09a (4)] found in the WIO region. Nevertheless, when comparing haplotypic diversity, South Africa comprised only three of the sixteen haplotypes currently known in the WIO. This suggests a small founder population for each species, probably supplied to southern African reefs from the tropics.

South African populations were also demographically unique, exhibiting high levels of ITS2 haplotype endemism compared to the rest of the WIO (Fig 4). Indeed, distinct structuring in South African *P*. *verrucosa* compared to lower latitude populations has been found [73]. It is also likely that the same patterns exist for all three SSHs in South Africa given their similar life histories. The genetic demography of South African *Pocillopora* suggests that recruitment at population and generational resolutions is self-sustaining. Our results support these findings [73], reporting *P*. *verrucosa* in southern Mozambique as a separate cluster from South Africa. This suggests weak connectivity between southern Mozambique and South African reef communities over ecological time scales. Similar patterns of diversity have been reported for other scleractinians in South Africa, such as *Acropora tenuis* [24] and other tropical taxa that demonstrate a southward attenuation in diversity [25]. The physical conditions of the Maputo Bay are generally unsuitable for the colonisation of large hermatypic populations and is a likely allotropic barrier to genetic connectivity between central Mozambique and South Africa. This biogeographic region probably affects the demography of these populations by suppressing population connectivity, possibly explaining the observed population indices.

We also report that nuclear DNA reveals some hybridisation between the *P*. *eydouxi* and *P*. *villosa* lineages. *Pocillopora verrucosa* exhibits negligible hybridisation with *P*. *eydouxi* and *P*. *villosa*. There is considerable evidence for high levels of hybridisation in marginal marine environments (20–23) and in *Pocillopora* (72,73), leading us to expect greater levels of hybridization than we observed. Causes for suppressed hybridisation are numerous, however, investigation of these mechanisms is beyond the scope of this study. Nevertheless, we can postulate mechanisms that may account for our observations: Firstly, pre- and post-zygotic barriers such as compatibility, genetic mules, and ecological fitness are common factors that determine the success of hybrids [19]. One-way introgressive hybridization of *Pocillopora* [74] may limit the possibility of numerous hybrid combinations. Secondly, ecological factors may play a role in inhibiting successful hybridisation. For example, *Pocillopora* communities on the MRC may resemble a functioning ecosystem more similar to those of lower latitude *Pocillopora* communities than anticipated [66]. Competition, larval development, niche space, or other factors may inhibit a suitable environment for sympatry of *Pocillopora* hybrids. Indeed, these are testable hypotheses and warrant further investigation.

## Conclusions

A recent drive to redress inconsistencies in *Pocillopora* classification has begun to align global congeners and taxonomic understanding. South Africa’s Maputaland Reef Complex (MRC) corals are influenced by its position at the southwestern margin of the Indian Ocean. The MRC harbours unique populations of *Pocillopora*, consisting of three to five species (considering sub-structuring within SSH09a) co-occurring throughout the southwestern Indian ocean, namely: SSH09a corresponding to *P*. *meandrina*/*P*. *eydouxi* morphotype (commonly misidentified as *P*. *verrucosa*), SSH13a corresponding to *P*. *verrucosa* morphotype (sometimes correctly identified, but also commonly misidentified as *P*. *damicornis sensu lato*), and SSH13b corresponding to the *P*. *villosa* morphotype (almost always misidentified as *P*. *eydouxi*). The results of this study demonstrate the possibility that previously investigated *Pocillopora* species in South Africa may have been misidentified. Nevertheless, due to the precision and consistency of taxonomy of these local studies [68,69,72,73], any incorrect taxonomy is likely to have little effect on conclusions, except for taxonomy based checklists [18,67]. We also report a unique assemblage of nuclear diversity on South African reef complexes. This diversity represents a unique but possibly fragile assemblage of *Pocillopora*, owing to the low observed genetic diversity and minimal hybridisation compared to the rest of the WIO. Considering these populations and their isolation from lower latitude populations and their demographic subsidies, this warrants the MRC’s protection and management. This study also demonstrates the universality of using the ORF marker for *Pocillopora* species identification and the importance of understanding the nature of species complexes at range margins.

## Supporting information

S1 Fig Phylogenetic analysis of *Pocillopora* sp. of South Africa in the Southwestern Indian Ocean (SWIO), using the part of D-loop and COI of the Control Region (CR). (A) Ultrametric phylogram of the two D-loop haplotypes. Node values indicate Ultrametric probability/ Bayesian probability/Maximum Likelihood values. Scale bar represents Millions of Years Before present. (B) Haplotype network coloured according to Primary Species Hypothesis (PSH) delineation and sized according to the number of individuals.
(TIF)

S2 Fig Phylogenetic analysis of *Pocillopora* sp. of South Africa in the Southwestern Indian Ocean (SWIO), using HSP70A. (A) Ultrametric phylogram of the 28 HSP70A haplotypes. Node values indicate Ultrametric probability/ Bayesian probability/Maximum Likelihood values. Scale bar represents Millions of Years Before present. (B) Haplotype network coloured according to Primary Species Hypothesis (PSH) delineation and sized according to the number of individuals.
(TIF)

S3 Fig Phylogenetic analysis of *Pocillopora* sp. of South Africa in the Southwestern Indian Ocean (SWIO), using the mtDNA region: Open Reading Frame (ORF) and part of the D-loop of the Control Region (CR). (A) Ultrametric phylogram of the concatenated haplotypes. Node values indicate Ultrametric probability/ Bayesian probability/Maximum Likelihood values. (B) Species delimitation methods using ABGD, ML PTP, B PTP, and GMYC methods using Primary Species Hypothesis (PSH) criteria, and Secondary Species Hypothesis (SSH) using thirteen microsatellite markers. C: Haplotype network coloured according to PSH delineation and sized according to the number of individuals. Scale bar represents Millions of Years Before present.
(TIF)

S4 Fig Phylogenetic analysis of *Pocillopora* sp. of South Africa in the Southwestern Indian Ocean (SWIO), using the nDNA region: ITS2 and HSP70A. (A) Ultrametric phylogram of the concatenated haplotypes. Node values indicate Ultrametric probability/ Bayesian probability/Maximum Likelihood values. (B) Haplotype network coloured according to Primary Species Hypothesis (PSH) delineation and sized according to the number of individuals. Scale bar represents Millions of Years Before present.
(TIF)

S1 Table General morphological characters for of *Pocillopora* sp. of South Africa in the Southwestern Indian Ocean (SWIO). Features of morphological characters presented here are strictly those that can be seen through general *in situ* and photographic observations.
(DOCX)
