# Supplementary figures and images for: Living on the edge: Assessing the diversity of South African Pocillopora on the margins of the Southwestern Indian Ocean
Source: PLoS One. 2019 Aug 2;14(8):e0220477. doi: 10.1371/journal.pone.0220477 (PMC6677312; doi:10.1371/journal.pone.0220477)

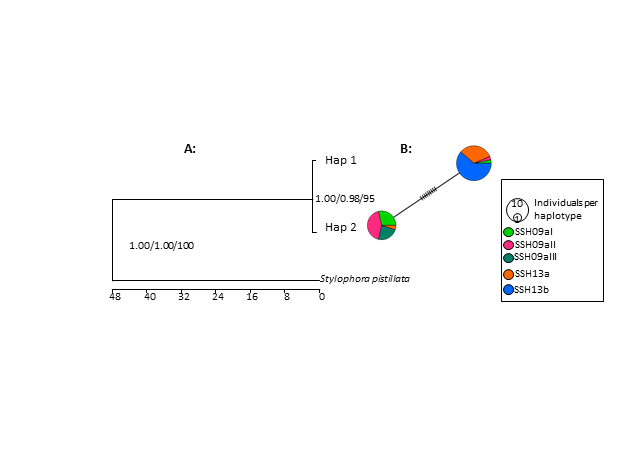

Supplement: S1 Fig — (A) Ultrametric phylogram of the two D-loop haplotypes. Node values indicate Ultrametric probability/ Bayesian probability/Maximum Likelihood values. Scale bar represents Millions of Years Before present. (B) Haplotype network coloured according to Primary Species Hypothesis (PSH) delineation and sized according to the number of individuals. (TIF) [file pone.0220477.s001.tif]

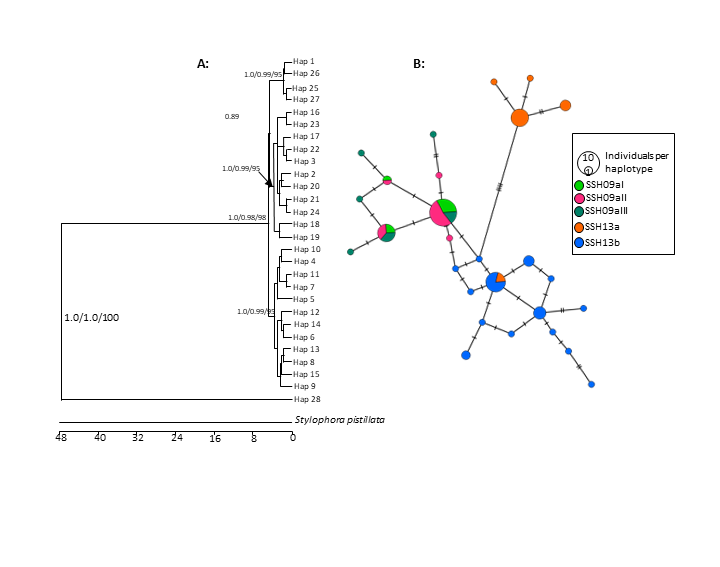

Supplement: S2 Fig — (A) Ultrametric phylogram of the 28 HSP70A haplotypes. Node values indicate Ultrametric probability/ Bayesian probability/Maximum Likelihood values. Scale bar represents Millions of Years Before present. (B) Haplotype network coloured according to Primary Species Hypothesis (PSH) delineation and sized according to the number of individuals. (TIF) [file pone.0220477.s002.tif]

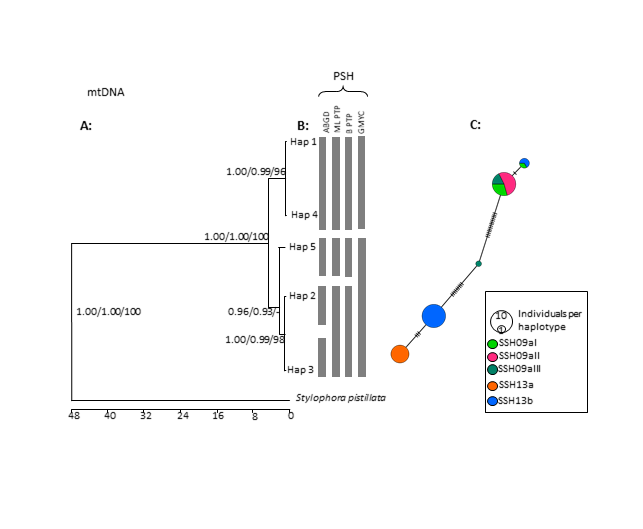

Supplement: S3 Fig — (A) Ultrametric phylogram of the concatenated haplotypes. Node values indicate Ultrametric probability/ Bayesian probability/Maximum Likelihood values. (B) Species delimitation methods using ABGD, ML PTP, B PTP, and GMYC methods using Primary Species Hypothesis (PSH) criteria, and Secondary Species Hypothesis (SSH) using thirteen microsatellite markers. C: Haplotype network coloured according to PSH delineation and sized according to the number of individuals. Scale bar represents Millions of Years Before present. (TIF) [file pone.0220477.s003.tif]

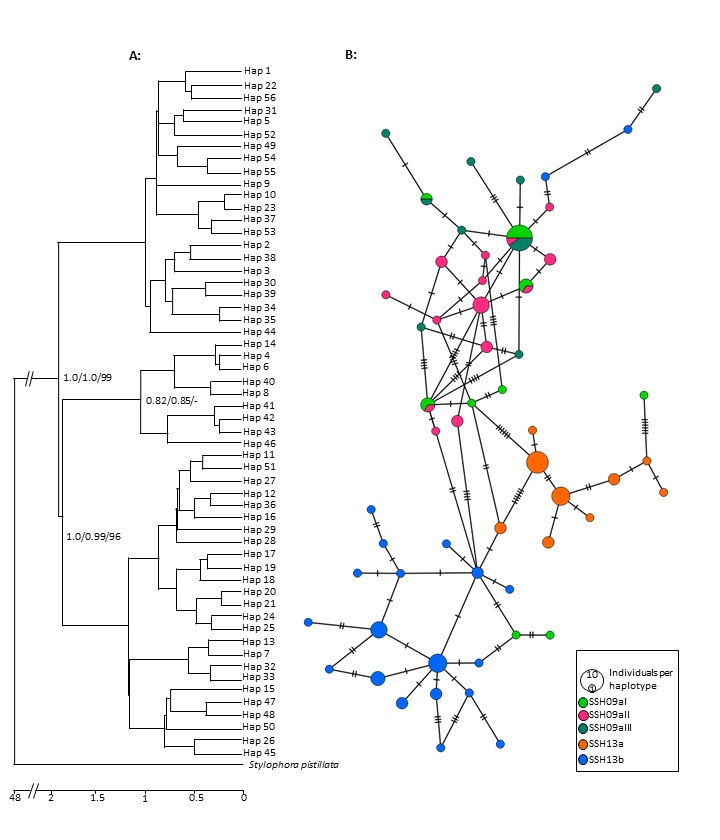

Supplement: S4 Fig — (A) Ultrametric phylogram of the concatenated haplotypes. Node values indicate Ultrametric probability/ Bayesian probability/Maximum Likelihood values. (B) Haplotype network coloured according to Primary Species Hypothesis (PSH) delineation and sized according to the number of individuals. Scale bar represents Millions of Years Before present. (TIF) [file pone.0220477.s004.tif]
