# Supplementary material for: Living on the edge: Assessing the diversity of South African Pocillopora on the margins of the Southwestern Indian Ocean
Source: PLoS One. 2019 Aug 2;14(8):e0220477. doi: 10.1371/journal.pone.0220477 (PMC6677312; doi:10.1371/journal.pone.0220477)
Supplement: S1 Table — Features of morphological characters presented here are strictly those that can be seen through general in situ and photographic observations. (DOCX) [file pone.0220477.s005.docx]

| Characters | Morphotype 1 | | |  | | Morphotype 2 | |  | Morphotype 3 |
| --- | --- | --- | --- | --- | --- | --- | --- | --- | --- |
|  | PSH09a | | |  | | SSH13a | |  | SSH13b |
|  | SSH09a1 | SSH09a2 | SSH09a3 | |  | |  |  |  |
| Verrucae | • Evenly spaced. • Neatly ordered. • Prominent, usually almost as wide as high. • Slightly longer than are wide. • Polyp extends to form more stony than velvety colony texture. | • Evenly spaced. • Neatly ordered. • Prominent, substantially longer than wide and protrude out and upward. • Polyp extends to form more stony than velvety colony texture. | • Evenly spaced. • Neatly ordered. • Prominent, usually almost as wide as high. • Slightly longer than are wide. • Polyp extends to form more stony than velvety colony texture. | |  | | • Irregular order and sometimes grade into each other. • Irregular size and shape. |  | • Evenly spaced with a close distribution. • Short and wide. • Polyps almost always extended, making a distinct velvet-like surface. |
| Branches | • Develop radially from a centre growth point, upright and laterally. • Thick and split once or twice within longer branches. • Split often and poorly defined to form paddle shape. • Large gaps between branches. • Irregular direction and branch shape. | • Develop radially from a centre growth point, mostly upright. • Thick, long and split once or twice near the base. • Large gaps between branches. • Somewhat regular direction and straight branch shape. | • Develop radially from a centre growth point, upright and laterally. • Thick and split once or twice in longer branches. • Split often and poorly defined to form paddle shape. • Large gaps between branches. • Irregular direction and branch shape. | |  | | • Stout and compact. • Closely knit. • Irregular cross-section profile. |  | • Thick and robust, often longer than 0.4m. • Cross section generally cylindrical, but can have club or paddle-shaped tips. • Branches often trifurcate from a point. • Large gaps between branches. |
| Colony | • Wider than high. • Generally radial in shape, but can have irregularly shaped footprint. • Usually not more than 0.5m high. | • Higher than wide. • Radial in footprint, usually strongly hemispherical.  • Usually 0.5m to 1m high. | • Wider than high. • Generally radial in shape, but can have irregularly shaped footprint. • Usually not more than 0.5m high. | |  | | • Develop radially but not always from a centre growth point. • Wider than high. • Generally radial in shape, but can have irregularly shaped footprint. • Usually less than 0.3m high, and 0.5m across. |  | • Always develop from a single point. • Radial footprint. • Hemispherical and often higher than wide. |
